# Heterogeneity of Environments Associated with Transmission of Visceral Leishmaniasis in South-Eastern France and Implication for Control Strategies

**DOI:** 10.1371/journal.pntd.0001765

**Published:** 2012-08-07

**Authors:** Benoit Faucher, Jean Gaudart, Francoise Faraut, Christelle Pomares, Charles Mary, Pierre Marty, Renaud Piarroux

**Affiliations:** 1 Laboratoire de parasitologie et mycologie, La Timone academic hospital, Marseille, France; 2 Aix-Marseille University, UMR-MD3, Marseille, France; 3 Aix-Marseille University, LERTIM EA3283, Marseille, France; 4 Service de Biostatistiques, La Timone academic hospital, Marseille, France; 5 Inserm U895, Université de Nice-Sophia Antipolis, Nice, France; 6 Service de Parasitologie–Mycologie, Hôpital de l'Archet, Centre Hospitalier Universitaire de Nice, Nice, France; Lancaster University, United Kingdom

## Abstract

**Background:**

Visceral leishmaniasis due to *Leishmania infantum* is currently spreading into new foci across Europe. *Leishmania infantum* transmission in the Old World was reported to be strongly associated with a few specific environments. Environmental changes due to global warming or human activity were therefore incriminated in the spread of the disease. However, comprehensive studies were lacking to reliably identify all the environments at risk and thereby optimize monitoring and control strategy.

**Methodology/Findings:**

We exhaustively collected 328 cases of autochthonous visceral leishmaniasis from 1993 to 2009 in South-Eastern France. Leishmaniasis incidence decreased from 31 yearly cases between 1993 and 1997 to 12 yearly cases between 2005 and 2009 mostly because Leishmania/HIV coinfection were less frequent. No spread of human visceral leishmaniasis was observed in the studied region. Two major foci were identified, associated with opposite environments: whereas one involved semi-rural hillside environments partly made of mixed forests, the other involved urban and peri-urban areas in and around the region main town, Marseille. The two neighboring foci were related to differing environments despite similar vectors (*P. perniciosus*), canine reservoir, parasite (*L. infantum* zymodeme MON-1), and human host.

**Conclusions/Significance:**

This unprecedented collection of cases highlighted the occurrence of protracted urban transmission of *L. infantum* in France, a worrisome finding as the disease is currently spreading in other areas around the Mediterranean. These results complete previous studies about more widespread canine leishmaniasis or human asymptomatic carriage. This first application of systematic geostatistical methods to European human visceral leishmaniasis demonstrated an unsuspected heterogeneity of environments associated with the transmission of the disease. These findings modify the current view of leishmaniasis epidemiology. They notably stress the need for locally defined control strategies and extensive monitoring including in urban environments.

## Author Summary

As *Leishmania infantum* was reported to be spreading in Europe, we conducted an exhaustive collection of visceral leishmaniasis cases in Provence-Alpes-Cô te d′Azur, the most active focus in France, from 1993 to 2009. The analysis of the 328 cases showed no spread inside the study area and a three-fold decrease of yearly incidence notably because cases associated with AIDS became less frequent. Distribution of the disease showed two distinct foci strongly associated with specific environments. One focus, close to the border with Italy, was associated with areas characterized by scattered habitation and mixed forest in the foothills as previously acknowledged. Oppositely, the other focus was centered in urban areas of Marseille. These results modify our view on the epidemiology of visceral leishmaniasis in Europe by highlighting the ability of the parasite to spread into urban environments. These findings stress the need for continuation of monitoring and prevention efforts and demonstrate that control strategy should be locally defined.

## Introduction

Visceral leishmaniasis (VL) due to *Leishmania infantum* remains a public health problem in the Mediterranean basin: despite underreporting, European reference centres record more than 400 cases each year [1]. Less frequently, cutaneous and mucosal manifestations may occur [2]. While overall VL incidence strikingly decreased since highly active antiretroviral therapy have been used to treat HIV infection [3], VL is currently emerging in several new foci, notably in Northern Italy [4]–[7]. Autochthonous animal infection was even reported in South Germany [5].

VL transmission requires that the parasite (*Leishmania infantum*), the sandfly vector (*Phlebotomus perniciosus* or *Phlebotomus ariasi* in France), the canine reservoir, and the human host meet [8]. In Mediterranean countries, such occurrence was reported to be strongly associated with specific rural environments [7] [9]: in the French rural focus of the Cevennes Mountains, *Leishmania* transmission by *P. ariasi* was showed 40 years ago to be associated with one ecological niche made of oak forest and chestnuts groves on the hillsides [10]. These findings were confirmed in other countries such as Morocco [11].

In South America, *L. infantum* VL epidemics were also reported in urban environments associated with building sites, garbage dumps, residual vegetation cover, and presence of various domestic animals such as rabbits, pigs and chicken [15]. In Europe, where sandfly species differ, urban transmission was reported notably in Athens, Lisbon, and Madrid [16]–[19].

The recent spread of *L. infantum* around the Mediterranean Sea was attributed to vegetation changes and movements of vectors or reservoir hosts due to global warming or to human activities [5] [6] [20] [21] whereas host factors such as the diffusion of new immunosuppressive treatments appeared marginal [22]. However, comprehensive studies about this suspected relation between environment and VL spread remain scarce despite calls for integrated monitoring [23] [24].

**Figure 1:**
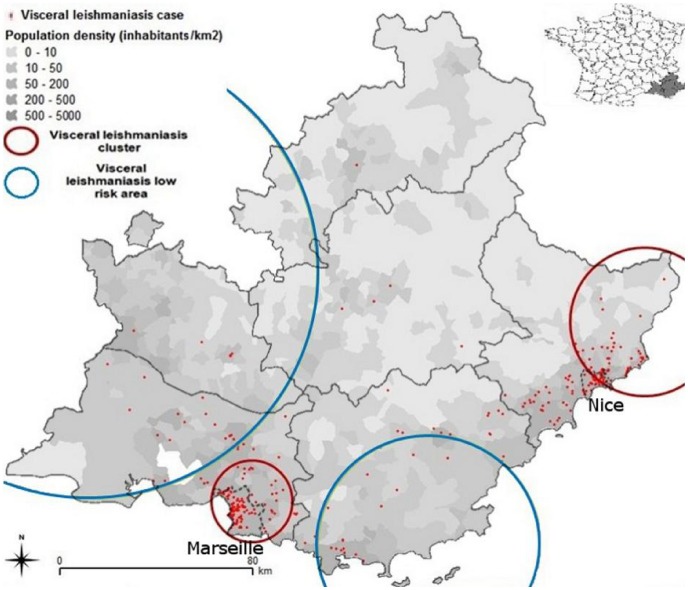
Visceral leishmaniasis clusters and low risk areas in Provence-Alpes-Cote d'Azur using SatScan.

Provence-Alpes-Cotes d'Azur (PACA) is a region covering 31,400 km2 in South-Eastern France inhabited by 4.500.000 people (figure 1). *Leishmania* transmission has been reported in PACA for 100 years [9]. Nowadays, PACA is the most active French VL focus: from 1999 to 2009, 132 of the 195 VL cases reported in mainland France occurred in PACA while the highest incidence numbers in France (6.6 VL cases per 1.000.000 inhabitants per year) were observed in the Nice Department (Figure 1) [25]. Besides, canine leishmaniasis has been spreading in PACA for the last ten years [26]. Only limited descriptions of the environments associated with VL transmission in PACA have been provided yet [9]. Specifically, none addressed urban transmission although VL was reported in the city of Marseille in the 1970s [27]. As PACA exhibits a wide range of Mediterranean natural environments including foothills as in the emerging VL focus in neighbouring Italy [7], it appeared to be a relevant area to study ongoing epidemiological trends. To allow optimization VL control strategies, we conducted this retrospective study over 17 years.

## Materials and Methods

### Objectives

The present study aimed to exhaustively collect cases of visceral leishmaniasis in PACA and test the hypothesis that the distribution of the disease was related to specific environments.

### Collection of cases

VL cases in PACA were exhaustively collected from 1993 to 2009. First, specific registries from the parasitological Departments of the two PACA academic hospitals (Marseille and Nice) were consulted. It is noteworthy that only these two laboratories perform *Leishmania* PCR and serology in PACA. Then, all departments of infectious diseases, general medicine, internal medicine, and pediatrics from the 81 PACA hospitals were contacted by phone to identify additional cases. After that, the microbiological laboratories of PACA hospitals were contacted by phone to look for missing cases. Finally, data obtained from Medical Information Departments of PACA hospitals enabled to confirm the consistency of the database. Cutaneous leishmaniasis, relapses and imported diseases were excluded. Age, gender, immunological status, time of diagnosis and place of residence were anonymously collected. Because our work did not imply any intervention (either diagnostic or therapeutic) but only relied on a retrospective collection of anonymous cases, we did not submit our research protocol to an ethical committee, in accordance with French laws.

### Geographical and environmental data

Geographical and environmental data included town boundaries and population, dog density, digital terrain model, wind resource potential, minimal temperatures, and land cover (using PACA CORINE land cover data obtained by comparing of remote sensing data [Landsat© images] and aerial pictures from 1999 and 2006 [www.eea.europa.eu/publications/COR0-landcover]). Land cover data was analysed using a 200 m wide buffer around places of residence. Land cover description was simplified to include the following 15 categories: 1) continuous urban area (i.e. buildings, roads and artificially surfaced area cover more than 80% of the ground, non-linear areas of vegetation and bare soil are exceptionally observed) 2) discontinuous urban area (i.e. buildings, roads and artificially surfaced area cover 50% to 80% of the ground, presence of non-linear areas of vegetation and bare soil); 3) scattered habitation; 4) industrial, commercial, and transport units; 5) mine, dump and construction sites; 6) green urban areas; 7) agricultural areas; 8) broad-leaved forest; 9) coniferous forest; 10) mixed forest; 11) transitional woodland/shrub; 12) moors and heathland; 13) open spaces without vegetation; 14) other natural spaces; 15) water bodies.

### Statistics

Spatial distribution of VL was first investigated using the Kulldorff's spatial scan statistic [28]. The SaTScan software (Kulldorf, Cambridge, UK, www.satscan.org) systematically moves a circular scanning window of increasing diameter over the studied region and compares observed case numbers inside the window to the numbers that would be expected under the null hypothesis (random distribution of cases). The maximum allowed cluster size corresponded to 50% of the population. The statistical significance for each spatial cluster was obtained through Monte Carlo hypothesis testing, i.e., results of the likelihood ratio were compared with 999 random replications of the dataset generated under the null hypothesis as recommended [29]. To avoid any misinterpretation due to methodological biases (mainly border effect and cluster shape effect), spatial clustering was also explored using SpODT (Spatial Oblique Decision Tree) [30]. This method, adapted from CART (classification and regression tree), builds oblique partitions of the study region providing spatial classes of homogeneous risk. Statistical significance was calculated using Monte Carlo inference as recommended.

Second, we investigated environmental characteristics underlying this spatial distribution. Univariate analysis was performed on environmental characteristics, using Fisher exact test. Because of the strong colinearity between these variables (prohibiting classical regression methods), the environmental characteristics were gathered in order to define environmental classes associated with VL. For that purpose, Multiple Correspondence Analysis (MCA) was carried out to generate an integrative description of the environments by defining a limited number of environmental classes. Hierarchical Ascendant Classification (HAC) was then performed to obtain the most homogeneous and the most distinctive classes (groups) according to similarity. The effect of the obtained classification on VL was tested using a logistic regression model. The absence of residual spatial autocorrelation of this final model was assessed by the Moran coefficient [29]. The analyses were all performed using R 2.11.1© (The R Foundation for Statistical Computing, 2009).

### Study design

The study was first conducted over the whole PACA region. Cases were linked to a georeferenced digitized map according to their home address using Quantum Gis 1.6.0®. The spatial distribution of VL was analysed by SatScan and SpODT using communal population numbers, i.e. all PACA inhabitants without reported VL were taken as controls. As environment study needed to be performed at an individual level, controls were then randomly selected from the 2008 telephone book: 1 control was selected per 10.000 inhabitants in each of the six departments of PACA without matching criterion. Environment around the places of residence of cases and controls was analysed as previously described using a 200 m wide buffer for land cover data extraction.

A specific study was then conducted focusing on the two regional main towns: Marseille (852,395 inhabitants) and Nice (347,060 habitants). To increase statistical power, additional controls were selected to obtain a ratio of two controls per one case. Spatial clustering and environmental risk factors were analysed as previously described.

## Results

### Demographic features

**Figure 2:**
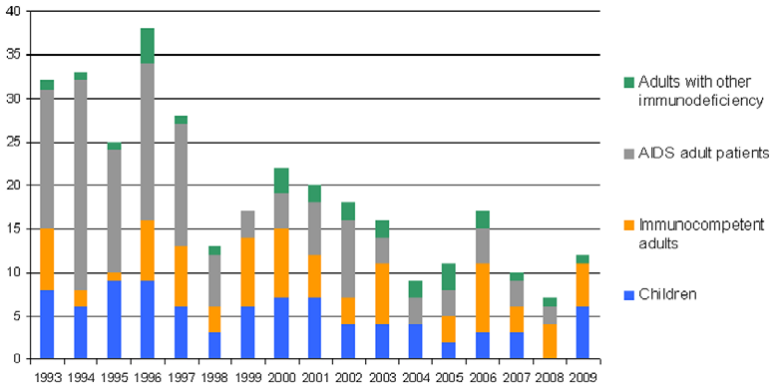
Visceral leishmaniasis cases diagnosed each year in Provence-Alpes- Cote d'Azur.

328 VL cases were collected (figure 2). Overall number of incident cases was 19.3 cases/year, decreasing from 31.2 cases/year between 1993 and 1997 to 11.4 cases/year between 2005 and 2009. Male were more often affected than female (220 cases, 67%), especially in case of HIV coinfection: 81% of HIV-infected patients were male. Median age was 36 years (0.4–90), with 87 patients (26.5%) under the age of 15 years including 73 (22.2%) under the age of five. One-hundred-sixty-two patients (49.4%) were immunodeficient, mostly because of HIV coinfection (133 cases, 40.5%). Immunodeficiency mostly affected adult VL patients (66%) but was rarely found in children (2%).

VL mean yearly incidence varied between the departments from 6/1,000,000 inhabitants in the Nice department neighbouring Italy to 0.4/1,000,000 in the mountainous northern department (Figure 1). The exact home address was obtained for 306 of the 328 collected cases (93.3%). In most cases, absence of address was due to a homeless status (14 cases) or because patients were leading a nomadic existence (2 cases).

### VL spatial distribution

SatScan results similarly showed a heterogeneous repartition (figure 1): two spatial clusters were identified accounting for 60.4% of cases. The most affected spatial cluster was located in a rural hillside area near Nice, the second regional main town. In this cluster, the main cities were affected only in the discontinuous urban areas or scattered habitations surrounding them. This cluster did not include the cities located closed to the seashore. These densely populated areas were indeed associated with significantly lower incidence numbers. This spatial cluster accounted for 64 cases (OR: 2.44, p<10^−9^). The second spatial cluster included as well continuous as discontinuous urban areas in and around Marseille. This spatial cluster included 116 cases (OR: 1.88, p<10^−5^). Between these two spatial clusters, a hilly area of intermediate risk included 78 cases. The rest of PACA was at low risk for leishmaniasis: Rhône River (valley and delta), coastal plains, and Alps Mountains (OR: 0.09, p<0.05). SpODT confirmed these results (p<10^−5^). Distribution did not differ between patients with and without HIV coinfection.

### Environmental characteristics analysis

Odds-ratios associated with specific environmental characteristics showed a contrast between the two spatial clusters according to univariate analysis (table 1): in the Nice spatial cluster, VL was significantly associated with scattered habitation and mixed forest; in the Marseille spatial cluster, VL was associated with the absence of agricultural areas.

**Table 1:** Significant association between risk of visceral leishmaniasis and environmental characteristics according to univariate analysis.

		Marseille focus		Nice focus	
Environmental characteristic	Category	OR (CI)	p	OR (CI)	p
Land cover: mixed forest	Presence	NS	NS	4.9 (2.2–11.8)	<10^−5^
Land cover: scattered habitation	Presence	NS	NS	2.8 (1.6–5.0)	<10^−3^
Land cover agricultural areas	Presence	0.5 (0.3–0.9)	0.02	NS	NS
Altitude			<0.01		<10^−5^
	<50 m^a^	1		1	
	50–300 m	2.2 (1.4–3.6)		3.7 (1.9–7.1)	
	300–1000	NS		3.3 (1.5–7.6)	
Slope			0.04		<10^−6^
	<15%^a^	1		1	
	15%–30%	2.7 (1.1–7.5)		3.6 (1.9–7.2)	
	>30%	NS		7.0 (2.8–19.3)	
Monthly minimum temperature			NS		<10^−3^
	>3°C^a^	NS		1	
	0–3°C	NS		3.1 (1.7–5.6)	
	<0°C	NS		NS	
Average wind velocity	High: 3.1–5 m/s	0.6 (0.3–0.9)	0.01	NS	NS

a taken as reference class for Odd-Ratio calculation.

NS: No significant difference, OR: Odd-Ratio, CI: 95% Confidence Interval.

**Figure 3:**
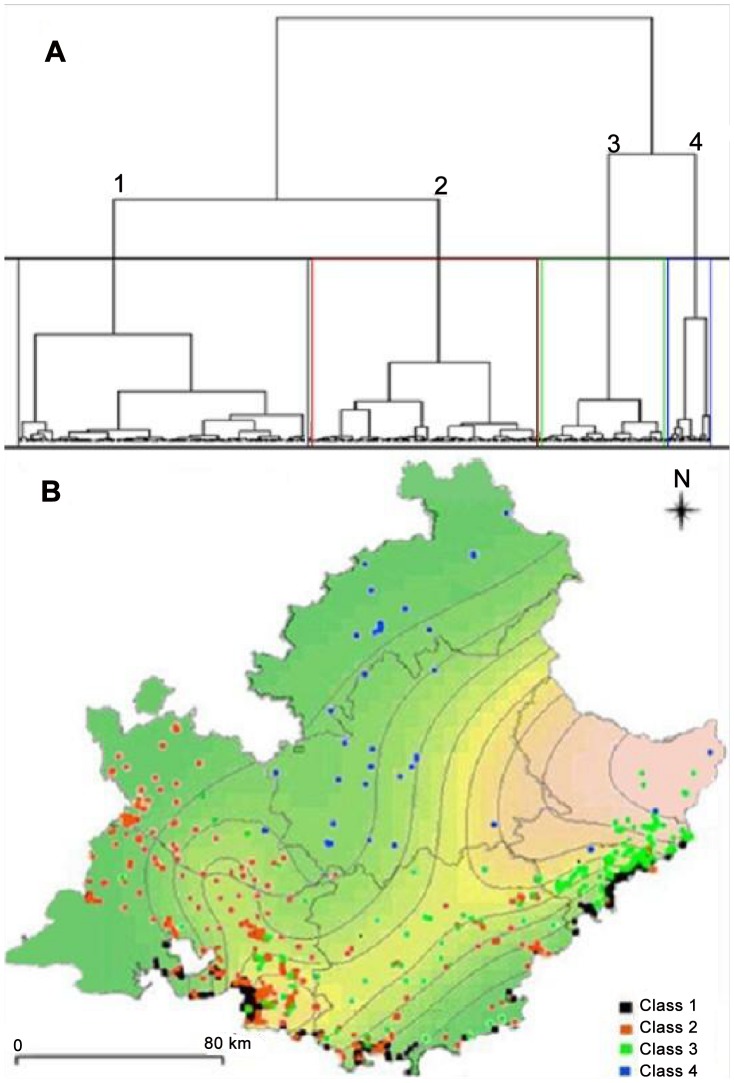
Environmental classes determined by multiple correspondence analysis. Hierarchical ascendant classification determined 4 environmental classes presented on a dendrogram (A) and on a map (B) of controls and visceral leishmaniasis cases produced using interpolation method based on spline functions [42].

Classification method (MCA) allowed identifying four environmental classes (Figure 3). The characteristics associated with each pattern are presented in Table 2. Numbers of cases and Odd-ratios associated with the various environment classes are presented in Table 3. Overall, the highest risk was associated with environmental class 3 associating scattered habitation, mixed forest, intermediate slope (15–30%), and intermediate monthly mean minimum temperature (0–3°C). An additional association was found in the Marseille focus between VL risk and environmental class 1 associating continuous urban area, absence of agricultural areas, low altitude (<50 m) and higher monthly mean minimum temperature (>3°C). Environmental class 1 was the most frequently found in VL cases in the focus in and around Marseille. Environmental classes explained VL distribution: when they were taken into account, no spatial autocorrelation was found anymore (Moran coefficient = 0.0039, p = 0.14).

**Table 2:** Main characteristics associated with the environmental classes determined by the hierarchical ascendant classification.

Environmental class	Main characteristics
Class 1	Continuous urban area
	Absence of agricultural areas
	Low altitude (<50 m)
	Higher monthly mean minimum temperature (>3°C)
Class 2	Intermediate monthly mean minimum temperature (0–3°C)
	High mean velocity of wind (3.1–5 m/s)
	Low slope (<15%)
	Presence of agricultural areas
Class 3	Scattered habitation
	Mixed forest
	Intermediate slope (15–30%)
	Intermediate monthly mean minimum temperature (0–3°C)
Class 4	Low monthly mean minimum temperature (<0°C)
	High (>300 m) and very high altitude (>1000 m)
	Scattered habitation

**Table 3:** Association between risk of visceral leishmaniasis and class of environment observed around the place of residence.

Environmental class	Whole region			
	Cases	Controls	OR (CI)	p
Class 1	113	185	1.9 (1.3–2.7)	<10^−3^
Class 2^a^	71	217	1	-
Class 3	116	54	6.6 (4.3–10.1)	<10^−15^
Class 4	7	31	NS	NS

	Focus north of Nice			
	Cases	Controls	OR (CI)	P
Class 1	37	69	NS	NS
Class 2^a^	1	7	1	-
Class 3	79	31	17.8 (3.0–341)	<10^−2^
Class 4	2	1	NS	NS

	Focus in and around Marseille			
	Cases	Controls	OR (CI)	P
Class 1	71	80	1.7 (1.1–2.6)	0.02
Class 2^a^	59	111	1	-
Class 3	16	5	6.0 (2.2–19.0)	<10^−3^
Class 4	0	0	-	-

a Class 2 was taken as reference class for Odd-Ration calculation.

NS: No significant difference, OR: Odd-Ratio, CI: 95% Confidence Interval.

### Urban analysis

**Figure 4:**
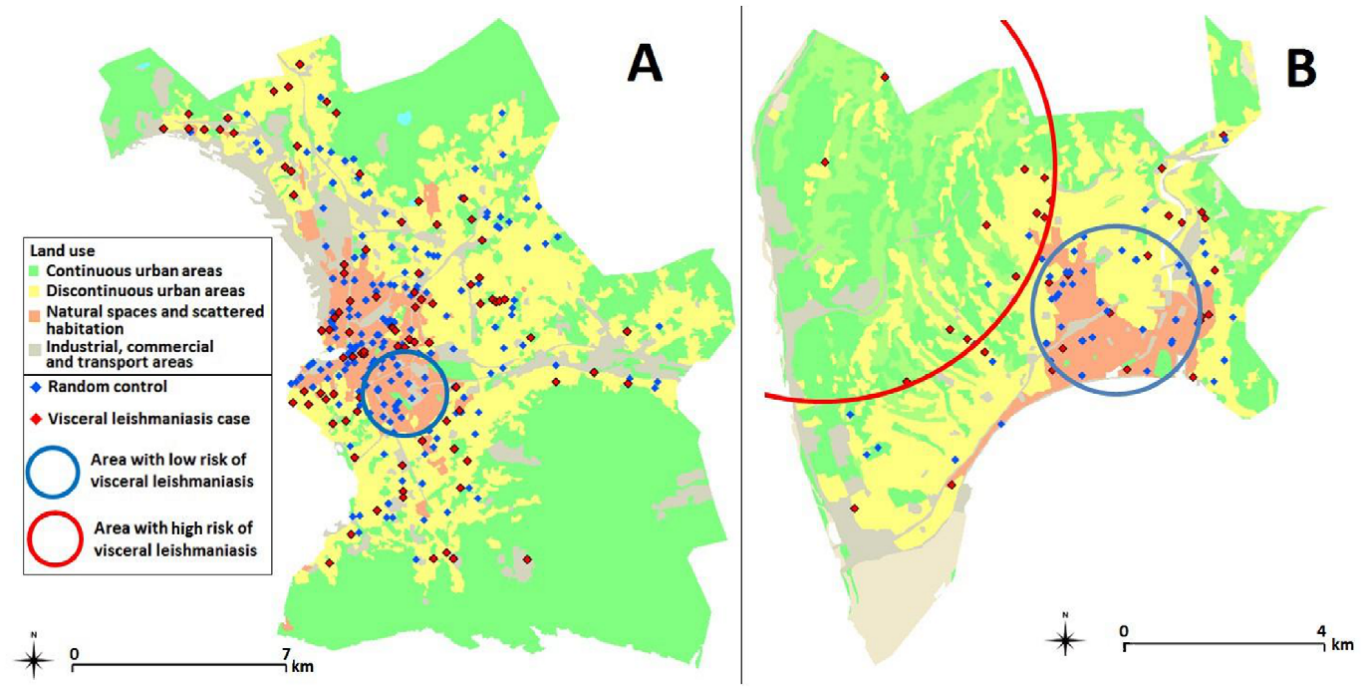
Visceral leishmaniasis high risk and low risk areas in Marseille (A) and Nice (B) using SatScan.

Distribution analysis using SatScan (Figure 4) and SpODT showed that, in Nice, VL cases were clustered in the foothills areas where there are no continuous urban areas (OR: 3.47, p<10^−2^) while they were significantly less frequently found downtown (OR: 0.27, p = 0.02). In Marseille, VL homogeneously involved most of the continuous urban areas of the city centre and surrounding discontinuous urban areas. Spatial distribution did not differ between patients with and without HIV coinfection.

Environment analysis similarly showed that VL risk was higher in Nice if scattered habitation (OR: 5.7 [1.4–27.8], p = 0.01) or mixed forest (OR: 15.5 [3.0–154.5], p<10^−3^) were observed near the place of residence. In Marseille, these associations were not observed.

## Discussion

This study benefits from several strengths. A large number of cases could be collected thanks to an excellent regional collaboration between 81 health facilities. Compared to the results of spontaneous reporting to the national reference centre [25], 27 additional cases could be identified between 1999 and 2009 (159 vs 132), illustrating the underreporting bias associated with passive monitoring methods. Because VL is a disease that always needs hospital settings to be diagnosed and treated, it can be assumed that the collection of cases was exhaustive or almost exhaustive. This enabled to rule out possible selection biases associated with passive collection of cases or thorough investigation focusing on limited territories. Additionally, the multiple geographical analyses enabled to assess for the first time the statistical significance of the observed clusters while ruling out a possible bias due to method specifications. Finally, the study design focusing on human diseases brought us to identify areas where the intensity of transmission led to a significantly higher incidence of human cases. The possible cases of infection far from the place of residence might have resulted in a loss of statistical power but they did not impact our study enough to prevent us from identifying significant clusters of cases. Though essential to define public health policies, such information could not be obtained from studies about canine leishmaniasis or asymptomatic carriage. Most human infections by *L. infantum* are indeed not associated with visceral leishmaniasis [8] [31] [32]. Our results are therefore complementary to those previously published.

The demographic features of present VL patients corresponded to previous descriptions [3] [25]. Specifically, pre-school children accounted for a minority of cases as usually in Europe, contrary to North Africa where VL mostly affects children under the age of three years [8]. Besides, almost half of the patients were immunodeficient, mostly because of HIV infection. Contrary to some regions such as northern Italy [4], VL incidence decreased in PACA since the 1990s. This overall decrease of VL incidence was largely related to a decrease of HIV/VL coinfections due to the availability of highly active antiretroviral treatments. Such evolution was observed in most European countries [3].

Our first finding of interest was that two limited foci of VL accounted for 2/3 of VL cases in PACA. These results modify our view of VL epidemiology in France, which is one of the main VL foci in Southern Europe [1]. Human VL foci appeared more limited in the current study than in previous reports based on a passive collection of human [33] or canine [26] leishmaniases. Contrary to what was observed in Italy [5] [7], no significant spread of human VL was found in PACA. Yet, a recent spread of canine leishmaniasis was reported in France [26]. This discrepancy suggests that human VL incidence was low in areas with recent introduction of L. infantum, highlighting the need for protracted monitoring. The monitoring system should therefore probably be based on mandatory rather than on spontaneous notification of human cases to increase its sensitivity, as differences in accuracy of passive and active monitoring were demonstrated by the 17% more cases identified with our active collection of cases compared to the spontaneous reporting to French National Reference Centre. However, the apparent spread of canine leishmaniasis might also be related to an improvement in the recognition and notification of canine cases as previous studies were based on unexhaustive collection of cases [26]. Overall, our findings did not confirm that human VL is currently spreading in PACA as it was observed in other European areas, notably in Italy.

Our results also revealed that VL transmission occurred in different environments in two foci though located 150 km apart despite identical parasite (*L. infantum* zymodeme MON-1), predominant vector (*P. perniciosus*), reservoir (dog), and human host [26] [33]. The focus north of Nice was associated with scattered habitation and mixed forest in the foothills as previously described [9]. Oppositely, the focus in and around Marseille was mostly associated with urban environment including continuous urban areas. The biology of *P. perniciosus* remains partly unknown [8] [34], but it was showed that *P. perniciosus* breeding sites can be found in heterogeneous biotopes from gaps among rocks to rubbish, basement and animal shelters which can explain the heterogeneous environments associated with VL transmission [34] [35]. The environmental differences between the two VL foci in PACA could be related to specific parasitic or vector subspecies. Because molecular studies proved able to distinguish sandflies on an infra-species scale [36], further entomologic studies might be of interest to investigate the vectors populations in these two foci. Previous publications did not report that such differing environments were associated with *L. infantum* transmission by *P. perniciosus* in France [9] [10] [11] [27]. A recent environmental risk mapping showed that VL transmission could occur in distinct environments in France, but it related each of them to a specific vector (i.e., *P. perniciosus* or *P. ariasi*) and failed to identify urban transmission [26]. Besides, sandflies were also found in northern territories where they sometimes caused canine leishmaniasis outbreaks [26]. This heterogeneity of involved environments is of major importance as current risk mapping strategies often rely on limited entomologic studies [24]. Results from retrospective studies about canine leishmaniasis in Europe confirmed that environment largely determined the distribution of canine leishmaniasis including in emerging foci [37]. These studies supported that heterogeneous environments were involved by showing that models based on overall data were less accurate than those based on local data.

Our results support the former hypothesis [10] that VL foci are distributed following the presence of vectors and not the density of the canine reservoir. Such result is worrisome as sandflies appeared to be spreading and might spread further North in France and in Central Europe. In particular, climatic conditions might become increasingly suitable because of global warming [21] [26]. However, this situation could change because of current campaigns advocating the use of deltamethrin-impregnated dog collars [38] and dog immunization [39]. In the future, VL distribution could depend on the frequency of their use as well as on the vector distribution.

The continuous urban transmission of VL in Marseille is a striking result in the current context of reported *Leishmania* spread [1] [5] [6], especially as it did not appear to be limited to areas with individual houses and important residual vegetal cover as reported in the 1970s [27]. A recent seroepidemiological study also described a homogeneous risk of *Leishmania* infection over the whole city of Marseille without predominance in discontinuous urban areas [40]. This result was also corroborated by the high rate of asymptomatic carriage found among Marseille healthy inhabitants [31]. This urban transmission was not observed in a recent study based on a passive collection of canine leishmaniasis cases in France [26] because Marseille veterinarians do not notify leishmaniasis cases to the national reference centre. Therefore, to allow setting up optimal monitoring and control strategies, awareness should be raised over the ability of *L. infantum* to fulfil its cycle in continuous urban areas.

Urban transmission was already incriminated in Athens, Greece [16], where it seemed to involve peri-urban environments made of discontinuous urban areas among quarries. This urban transmission in downtown Athens appeared of lower intensity than that observed in Athens suburbs according to a study of canine seroprevalence [17] contrary to our findings in Marseille. Urban transmission was also observed in Madrid, Spain, where canine seroprevalence was as high (around 5%) in peri-urban than in rural areas [18]. In Lisbon, Portugal, presence of infected vectors was demonstrated inside the city and canine seroprevalence appeared to increase from 5.5% in 1980 to 19.2% in the early 2000s [19], raising concerns about a progressive increase of VL transmission in the city. In Italy, *P. perniciosus* was observed in new residential urban districts [41]. All these studies did not allow for tracing of transmission to downtown rather than peri-urban environments, and mostly focused on canine leishmaniasis which is much more widely distributed than human VL.

The specific environments associated with a higher risk of VL transmission in the Marseille urban focus need to be further investigated. The negative correlation with higher wind velocity was unsurprising because sandflies do not easily fly in case of wind [8] [36]. Similarly, the apparent lower VL risk associated with agricultural areas around Marseille could be related to mechanical or chemical destruction of sandflies' breeding sites [8]. However, these associations were not confirmed by multivariate analyses and should therefore not be overinterpreted. Besides, these associations were not observed in the Nice focus. Interestingly, most affected areas in Marseille were located inside the perimeter of a major city renovation project. *P. perniciosus* breeding sites were previously found in abandoned buildings and in animal shelters such as those of watch dogs [34] and the numerous rats observed in these areas were sometimes suggested to be a possible reservoir [35]. Besides a higher risk of VL associated with construction and waste sites was described in South America [12] [13] [14] but such result cannot be extrapolated to Europe because vectors differ. Identifying the environments associated with this urban transmission is all the more important as response strategy based on environmental vector controls proved effective elsewhere [13].

As a conclusion, the use of new geographical and statistical tools allowed revisiting the close relation between parasite transmission and environment and thereby improving our understanding of VL epidemiology. While the strong link between VL risk and the previously incriminated environment was confirmed, it was found that VL could indeed involve other environments including continuous urban areas. These results raise concern about a possible underestimation of the current and future spread of *L. infantum* around the Mediterranean Sea. By suggesting the risk of a higher future burden than previously expected, our findings plead for the continuation of current strategies for control as those taking place in the current European program EDENext (www.edenext.eu). Our results specifically underline the need for local definition of control strategies and for extensive monitoring including in urban environments.
